# Pericarditis Linked to the Development of an Anterior Mediastinal Cholesterol Granuloma

**DOI:** 10.1016/j.atssr.2023.01.003

**Published:** 2023-01-25

**Authors:** Omar Toubat, Kimberly A. Shemanski, W. Dean Wallace, Anthony W. Kim

**Affiliations:** 1 Department of Surgery, Keck School of Medicine, University of Southern California, Los Angeles, California; 2 Department of Surgery, University of Virginia, Charlottesville, Virginia; 3 Department of Pathology, Keck School of Medicine, University of Southern California, Los Angeles, California

## Abstract

Cholesterol granulomas are benign, foreign body giant cell reactions that occur in response to the presence of cholesterol crystals. Cholesterol granulomas are an exceedingly rare entity in the mediastinum and are often found incidentally without a known cause. In this case report, we describe a cholesterol granuloma developing in the anterior mediastinum secondary to a prolonged episode of pericarditis. In addition to providing unique insights into the etiology of cholesterol granulomas of the anterior mediastinum, this report also demonstrates the feasibility of robotic surgical approaches for the management of these lesions.

A cholesterol granuloma is a benign, foreign body giant cell reaction that occurs in response to the presence of cholesterol crystals.^1^ Although commonly described in the mastoid process or middle ear space, cholesterol granulomas are an exceedingly rare entity in the mediastinum and are often found incidentally without a known cause. Here, we provide a novel report of a cholesterol granuloma developing in the anterior mediastinum secondary to a prolonged episode of pericarditis that was managed by a robotic thoracic surgical approach.

The patient is a 79-year-old woman who was incidentally found to have a 2.1 × 1.1 × 1.3-cm spiculated nodule in the right middle lobe (RML) and 1.1 × 1.4-cm soft tissue calcification in the anterior mediastinum on chest computed tomography (CT) while undergoing evaluation for persistent pericarditis (Figures 1A, 1B). After resolution of pericarditis with a course of colchicine and indomethacin, subsequent positron emission tomography (PET)/CT scan demonstrated 18-fluoro-2-deoxyglucose (FDG) hyperactivity in both the RML nodule (standardized uptake value [SUV], 2.7) and the thymus (SUV, 9.6; Figures 1C, 1D). The patient is a former smoker with a remote 2.5 pack-year history and a positive family history of lung cancer in her sister. Biopsy of the spiculated RML nodule demonstrated chronic inflammation and organizing pneumonia with nonnecrotizing granulomas but no evidence of malignant disease. The patient elected to undergo imaging surveillance of the anterior mediastinal lesion. The size of the mediastinal lesion remained stable on interval CT imaging performed 4 months after initial presentation, and it did not demonstrate elevated metabolic activity on PET dotatate imaging, suggesting that it was not of neuroendocrine etiology. Six months after her initial presentation, the patient began to have symptoms of increasing chest pressure behind the manubrium and elected to proceed with an operation for removal of the anterior mediastinal mass.

**Figure 1:**
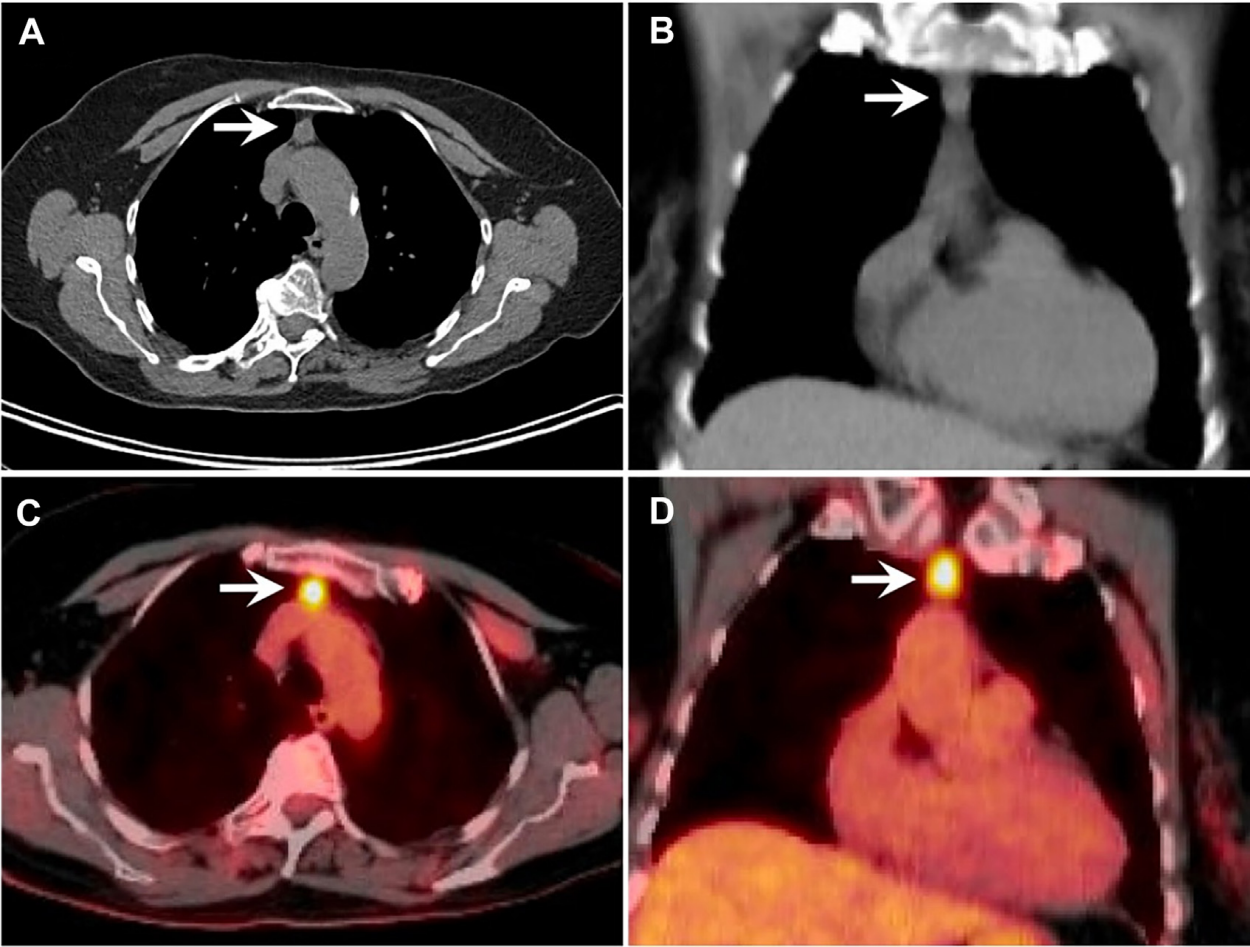
(A, B) Preoperative non–contrast-enhanced chest computed tomography scan demonstrates soft tissue calcification in the anterior mediastinum (arrow) with (C, D) positron emission tomography/computed tomography imaging showing metabolic hyperactivity in the thymus (arrow).

The patient underwent robot-assisted thymectomy. The thymus was completely mobilized from surrounding structures and resected without complication. There were no particular challenges to the operation due to the history of pericarditis. After the thymectomy, the RML was explored in an attempt to identify the previously described benign spiculated nodule. After an extensive exploration, no lesion could be identified in the RML, and the decision was made to close. The patient tolerated the operation well, which went without complications. Her postoperative course was unremarkable, and she was discharged home on postoperative day 2. The gross thymus specimen is shown in Figure 2A. Final pathologic examination demonstrated a xanthogranulomatous nodule with numerous cholesterol clefts, consistent with a benign cholesterol granuloma (Figures 2B, 2C). The patient remained asymptomatic 44 months after the operation. The most recent chest CT imaging shows a stable 2.0 × 0.6-cm RML opacity localized to the site of the previously biopsied spiculated nodule.

**Figure 2:**
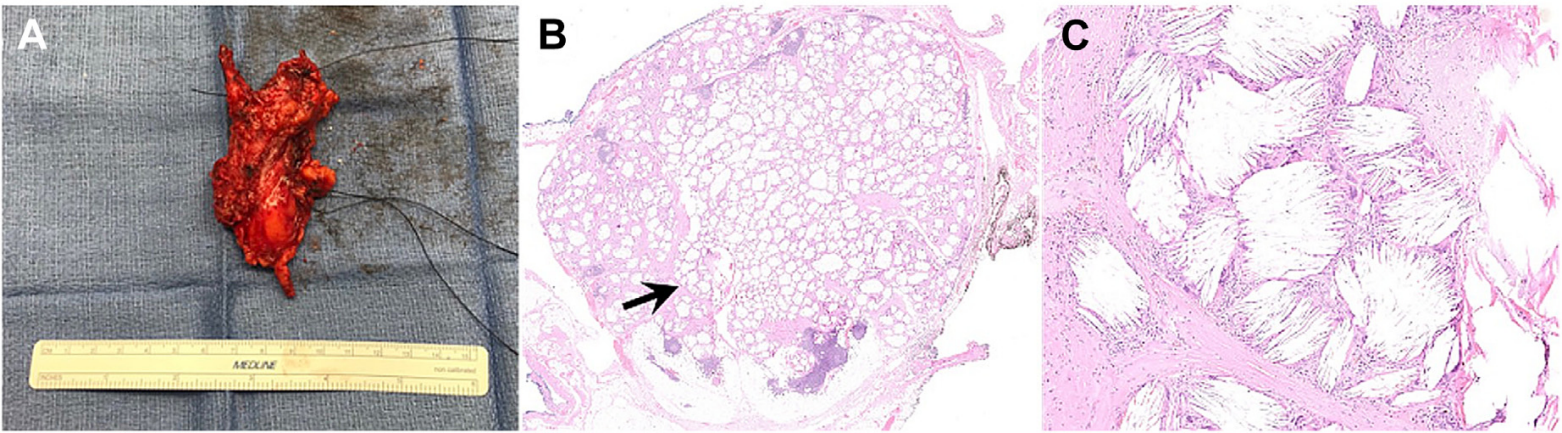
(A) Gross thymus specimen demonstrates a yellow-tan lobulated fibroadipose tissue measuring 8 × 5.5 × 1.2 cm. (B) Low-power hematoxylin and eosin stain of the cholesterol granuloma reveals well-circumscribed and nodular shape (arrow) magnified in C. (C) High-power view of cholesterol granuloma showing numerous clusters of cholesterol clefts surrounded by foreign body giant cells.

## Comment

Cholesterol granulomas of the anterior mediastinum are rare, with only a handful of reports described in the literature.^1^, ^2^, ^3^, ^4^, ^5^, ^6^, ^7^ These benign processes originate from a foreign body giant cell reaction to the presence of cholesterol crystals. This report describes an anterior mediastinal cholesterol granuloma developing secondary to pericarditis and subsequently managed robotically.

The exact mechanism of cholesterol granuloma formation is unclear as these lesions are often identified incidentally. It is hypothesized that such lesions develop secondary to local trauma or inflammation.^1^^,^^2^^,^^4^ This patient experienced a prolonged course of symptomatic pericarditis for 3 months before the identification of the mediastinal lesion. It is both biologically plausible and temporally consistent that this episode of pericarditis served as the inciting cause of the anterior mediastinal cholesterol granuloma formation.

This report describes PET FDG hyperactivity localized to the site of the granuloma, consistent with the active inflammatory process present in such lesions. Similar PET/CT findings have been reported in other studies.^5^^,^^7^^,^^8^ However, PET FDG hyperactivity can also be observed in more common pathologic processes of the anterior mediastinum, including primary lung cancers, metastatic lesions, and thymomas. In this case, SUV avidity of the cholesterol granuloma was less than would be expected in malignant pulmonary or metastatic lesions yet greater than would be expected for a thymoma. Therefore, although the presence of enhanced FDG uptake alone has limited diagnostic utility in a thymic cholesterol granuloma, the level of SUV avidity may be an important diagnostic indicator of this disease process. Conversely, poor uptake of novel PET radioisotopes such as dotatate may be used to rule out neuroendocrine tumors and further assist in radiographically narrowing the differential diagnosis.

Given the challenges associated with radiographic assessment and diagnosis of mediastinal cholesterol granulomas, one may argue for preoperative tissue sampling in this setting. In this case, the small, retrosternal location of the mediastinal mass precluded the opportunity for biopsy. Prior reports have commented on the limitations of mediastinal biopsies, including poor specimen yields and the difficulty in differentiating mediastinal cholesterol granulomas with scattered cholesterol clefts observed in more common disease processes.^6^ In addition, it is recommended that patients undergo preoperative tissue biopsy if the mediastinal mass is unlikely thymic in origin or if the suspected thymoma or thymic carcinoma appears locally advanced or unresectable. As in this report, if the thymic mass appears surgically resectable, it is appropriate to proceed with surgical resection without biopsy to maintain capsular integrity. Ultimately, reports such as ours reaffirm the importance of surgical excision and histopathologic assessment in the diagnosis of anterior mediastinal cholesterol granulomas. Complete surgical resection is also curative in patients with symptomatic disease related to the space-occupying effects of the lesion.

In conclusion, we present a novel case of a cholesterol granuloma of the anterior mediastinum developing secondary to pericarditis. This report reaffirms the importance of including cholesterol granulomas in the differential diagnosis of anterior mediastinal masses, especially with an antecedent history of an inflammatory disorder. Furthermore, this case demonstrates that despite an inflammatory disorder, a robot-assisted surgical approach in the diagnostic and therapeutic management of these benign lesions is feasible.
